# Alterations in Beta Cell Identity in Type 1 and Type 2 Diabetes

**DOI:** 10.1007/s11892-019-1194-6

**Published:** 2019-08-10

**Authors:** Abu Saleh Md Moin, Alexandra E. Butler

**Affiliations:** Diabetes Research Center, Qatar Biomedical Research Institute, Hamad Bin Khalifa University, Qatar Foundation, PO Box 34110 Doha, Qatar

**Keywords:** β Cell, Dedifferentiation, Transdifferentiation, Type 1 diabetes, Type 2 diabetes, Pancreas

## Abstract

**Purpose of Review:**

To discuss the current understanding of “β cell identity” and factors underlying altered identity of pancreatic β cells in diabetes, especially in humans.

**Recent Findings:**

Altered identity of β cells due to dedifferentiation and/or transdifferentiation has been proposed as a mechanism of loss of β cells in diabetes. In dedifferentiation, β cells do not undergo apoptosis; rather, they lose their identity and function. Dedifferentiation is well characterized by the decrease in expression of key β cell markers such as genes encoding major transcription factors, e.g., MafA, NeuroD1, Nkx6.1, and Foxo1, and an increase in atypical or “disallowed” genes for β cells such as lactate dehydrogenase, monocarboxylate transporter MCT1, or progenitor cell genes (Neurog3, Pax4, or Sox9). Moreover, altered identity of mature β cells in diabetes also involves transdifferentiation of β cells into other islet hormone producing cells. For example, overexpression of α cell specific transcription factor Arx or ablation of Pdx1 resulted in an increase of α cell numbers and a decrease in β cell numbers in rodents. The frequency of α-β double-positive cells was also prominent in human subjects with T2D. These altered identities of β cells likely serve as a compensatory response to enhance function/expand cell numbers and may also camouflage/protect cells from ongoing stress. However, it is equally likely that this may be a reflection of new cell formation as a frank regenerative response to ongoing tissue injury. Physiologically, all these responses are complementary.

**Summary:**

In diabetes, (1) endocrine identity recapitulates the less mature/less-differentiated fetal/neonatal cell type, possibly representing an adaptive mechanism; (2) residual β cells may be altered in their subtype proportions or other molecular features; (3) in humans, “altered identity” is a preferable term to dedifferentiation as their cellular fate (differentiated cells losing identity or progenitors becoming more differentiated) is unclear as yet.

## Introduction

Both classic forms of diabetes mellitus are characterized by the inability of pancreatic β cells to meet the demand of insulin secretion due to either a nearly complete loss (type 1 diabetes [T1D]) or a deficit of functional β cells in the setting of peripheral insulin resistance (type 2 diabetes [T2D]). The deficit in β cell mass (~ 90% in long-standing T1D [1], ~ 65% in long-standing T2D [2]) has long been proposed to be a consequence of β cell death. However, this principle has recently been challenged by studies mainly performed in animal models, with the suggestion that, rather than β cell death, the deficit in β cell mass is due to β cell dedifferentiation or transdifferentiation [3••, 4].

Functional β cell mass refers to adequate numbers of appropriately functioning β cells; a deficit in either number or function/identity can therefore lead to a diminution of “functional β cell mass” [5, 6]. Altered β cell identity, rather than β cell apoptosis, in the setting of chronic hyperglycemia was first reported in Sprague-Dawley rats [7]. Subsequently, others reported a loss of mature β cell identity accompanied by dedifferentiation in the diabetic state [3, 8••, 9••, 10••]. Upon dedifferentiation, β cells regress to a less mature or even precursor-like state, leading to a loss of key components responsible for optimal performance, most notably in terms of insulin secretion. However, there is ambiguity in defining “dedifferentiation,” as altered β cell identity can also be explained as “β cell degranulation,” where insulin granules are depleted due to metabolic stresses [9••, 11]. Emerging evidence also suggests that phenotypic alterations of β cells can promote transdifferentiation to other pancreatic endocrine cell types (mainly α cells and δ cells), a phenomenon that has been observed in both diabetic animals and human subjects [4, 9••, 10••, 12, 13]. Regardless of how β cell dedifferentiation is defined, its importance in diabetes research stems from the direct implications for β cell function, turnover, and regeneration. In this review, we will discuss the characteristics of human pancreatic β cells, the concept of “β cell identity-crisis,” and the factors underlying the altered identity of β cells in diabetes.

## Fetal Development of Human Endocrine Pancreas: the Transcriptional Roadmap of Human β Cell Differentiation

The organogenesis of human pancreas involves regulation at the level of gene transcription to furnish mature pancreatic cells. Originating from a pool of apparently identical progenitor cells, the mature pancreas comprises endocrine, exocrine, and ductal cell types that collectively synthesize and secrete the hormones and enzymes required for nutritional homeostasis [14]. The endocrine compartment (Islets of Langerhans) further differentiates into five cell types (α, β, δ, PP, ϵ) that have obvious similarities in expression of common genes and ability to secrete hormones [14]. In comparison to rodent pancreas, the knowledge of gene expression profiles during early human pancreas development is limited. However, several studies suggest the involvement of conserved genetic regulatory networks and transcription factors [14–16].

The pancreas originates from two primary diverticula of the primitive gut at 26-day post-conception (dpc; ~ 6-week gestation) [15]. After gastrulation, the earliest event leading to pancreas development is exclusion of sonic hedgehog (SHH) signaling from the dorsal endoderm where it is in contact with the notochord [17], which allows expression of the key transcription factor pancreatic and duodenal homeobox factor 1 (PDX1) [18, 19]. The gut epithelium evaginates into the surrounding mesoderm-derived mesenchymal tissue from the dorsal and ventral pancreatic buds, which at Carnegie stage 13 (CS13, ~ 6.5-week gestation) in humans is marked by the transcription factors SRY (sex determining region Y)-box 9 (SOX9), PDX1, and GATA binding protein 4 (GATA4) [14, 15]. The buds expand, branch, and fuse, and, by CS15 (~ 7.5-week gestation), gut rotation brings the buds together on either side of the portal vein [14, 20], the ventral portion giving rise to the lower head of the pancreas and the dorsal portion giving rise to the body, tail, and upper head portion of pancreas. Between CS15 and CS19, the central duct-like structure (“trunks”) contains a pool of multipotent progenitor cells that express more SOX9/NKX6.1 but less GATA4, whereas the more peripheral clustered cells (“tips”) are triple-positive (SOX9/GATA4/NKX6.1) [14]. By CS19 (~ 9-week gestation), the pancreatic progenitor cells become more differentiated, with segregation of acinar and endocrine precursors.

Endocrine pancreas formation requires the transient activation of Neurogenin3 (NEUROG3), as NEUROG3^+^ cells generate the five endocrine cell subtypes. NEUROG3 expression increases rapidly during late embryogenesis and is closely linked to the appearance of fetal insulin (CS20–21) (~ 9–10-week gestation) [14]. Fetal β cells demonstrate a unique transcription factor signature (for example, loss of SOX9) with subsequent detection of nuclear NKX2.2, NKX6.1, PDX1, FOXA2, ISL1, and insulin [14]. Co-expression of several hormones is notable amongst endocrine cells in the developing human pancreas. Polyhormonal cells are typically present either as single cells or in small clusters in the acinar parenchyma, suggesting that these cells represent newly forming islets [21–23].

Formation of secretory granules with an increase in insulin content occurs in β cells at ~ 10–14-week gestation [24], a concept supported by the expression of more differentiated β cell markers (PC1/3, IAPP) in virtually all β cells at 12–14 weeks of development [15, 25]. Data from previous studies suggests that large islets of mixed types (akin to adult islets) are formed only after 21-week gestation [21] and are accompanied by increased numbers of β cells, produced by β cell proliferation, thereby increasing the β/α and β/δ cell ratios [26]. After birth, the human pancreatic β cell proliferation index is highest during the first 2 years, gradually decreasing until, after the age of 3–5 years, replication of existing β cells is negligible in most cases [27].

## Postnatal Distribution and Identity of Mature β Cells

To study β cell dedifferentiation, it is important to understand how pancreatic β cells are organized in the complex cytoarchitecture of an islet (reviewed in [28]). Detailed quantitative studies have revealed that human pancreatic islets consist of ~ 60% insulin-producing β cells and 30% glucagon-producing α cells, the remaining 10% consisting of δ cells (somatostatin), γ cells (PP) and ϵ cells (ghrelin) [29–31] randomly distributed throughout the islet [32, 33]. Exactly how this architecture affects cell-to-cell interactions leading to regulated and concerted hormonal secretion remains to be determined; however, a modified core-mantle structure of the human islet has been proposed where, in small human islets (40–60 μm in diameter), β cells are in the core position surrounded by α cells in the mantle position, with a more complex intermingled arrangement found in larger islets [34]. Similar studies also reported that β cells are contiguously arranged either into smaller clusters surrounded by non-β cells or along invaginations of the exterior surface of the islet [35, 36]. An alternative arrangement of β cells in human islets has also been described with β cells intermingling freely (without clustering) with other endocrine cells [30]. The unique topological arrangements of β cells (especially β-β cell contacts) in human islets has measurable consequences in terms of islet function [37, 38]. In addition to intra-islet connections, individual human β cells and larger aggregates of cells (in association with the mesenchymal protein vimentin) can form within the ductal epithelium and migrate during gestation, suggesting that β cells are capable of remodeling [39]. In humans, β cells harbor connexin-36 (Cx36) gap junctions that create channel coupling between β cells, and which correlate with insulin secretion, suggesting the dependence of functional identity of human β cells on gap junction coupling [40].

A β cell is classically defined by its function: synthesizing, sorting, and secreting insulin. However, the hallmark of a mature β cell involves a more complex cellular identity with finely tuned coupling to the prevailing glucose level. Ultrastructurally, β cells are marked by the presence of electron dense-core insulin granules with a clear peripheral mantle (size, ~ 300 nm and number, ~ 10,000 per β cell). The presence or absence of fully processed proinsulin molecules in these dense-core granules depends on the maturity of the β cell [41–43]. As previously noted, the fate of islet endocrine cells is decided by the ON/OFF switch and concerted activities of key transcription factors during development; mature β cells are also distinct in terms of expression of certain genes and transcription factors. Single-cell analyses that have revealed the transcriptional program of human pancreatic endocrine cells depicted genes (for example, PAX4, PDX1, MAFA, MAFB, DLK1, SIX2/3, ID1, IAPP, UCN3, OLIG1) that are highly or exclusively expressed in human β cells ( [44••] and reviewed in [45]). Expression of certain genes has revealed notable cell-type and species differences. For example, in humans, MAFB expression has been detected in adult α and β cells [44••, 46••], whereas MafB and MafA expressions become restricted to α and β cells, respectively, in the mouse [47, 48]. In addition, SIX2 and SIX3, two recently identified transcription factors reported in human β cells [46••, 49, 50••], have been shown to enhance insulin content and secretion in immature β cells, suggesting their crucial role in human β cell maturation [51].

Heterogeneity of β cells has recently received increasing attention, as emerging evidence (mainly from rodent studies) suggests that β cells pass through different maturation states in adult islets [52•, 53••]. During development, distinct subpopulations of β cells have been observed [54, 55•]. In adult human islets, marker analysis coupled with single-cell RNA sequencing (scRNA-seq) revealed two surface markers, ST8SIA1 and CD9, that discriminated four distinct human β cell subpopulations (β1–4), each expressing common β cell markers but displaying differences in insulin secretion rates and gene expression profiles [56••]. It is however uncertain whether in adults these subsets are proportionally stable or whether they can interconvert. A population of β cells has been shown to develop during the progression of T1D in non-obese diabetic (NOD) mice which have characteristics of immature proliferation, reduced insulin granule content, and reduced functional capacity with relative resistance to cell death [57]. In T2D, three distinct subpopulations of β cells were identified, shifting in number with age or BMI [58••], suggesting a possible correlation of β cell heterogeneity and dysfunction in diabetes and aging, due partly to altered phenotypes of human β cells in islets. Apart from the molecular heterogeneity, functional heterogeneity within the β cell population also exists which might offer advantages for the ability of β cells to respond robustly to different physiological conditions. For example, by utilizing a set of novel techniques, insulin secretion responses were found to be orchestrated by two populations of β cells in rodents: the hub cells, which function as pacemakers to control the insulin secretion dynamics, and the follower cells that respond to hub cell signaling cues [59••]. More detailed molecular characterizations of subgroups of β cells are required to determine their biological significance, function, ontogeny, and involvement in disease. The main challenge in this regard is the high donor-to-donor variation identified by single-cell studies [46, 52•, 58••]; however, a recently developed single-cell heterogeneity analysis algorithm (RePACT) might help to resolve these issues and to identify β cell specific disease genes [60•].

β Cell turnover is a critical factor for maintaining functional β cell mass in health. Postnatal β cell mass is dynamic and maintained by the balance of cell birth/renewal (by replication of pre-existing differentiated β cells and by neogenesis or differentiation from progenitor cells) and cell death (usually by apoptosis) [27, 61]. Though adult β cells are largely in a quiescent state (evidenced by a very low frequency of proliferation) [27], β cell mass demonstrates a degree of plasticity, expanding in response to metabolic demands associated with the insulin resistance of pregnancy [62] and obesity [2]. The establishment of β cell mass during childhood likely plays a crucial role in successful or failed adaptation to increased β cell metabolic workload demand, a key factor for the onset of T2D [63, 64].

## Dedifferentiation of Pancreatic β Cells—Altered Identity

**Fig. 1:**
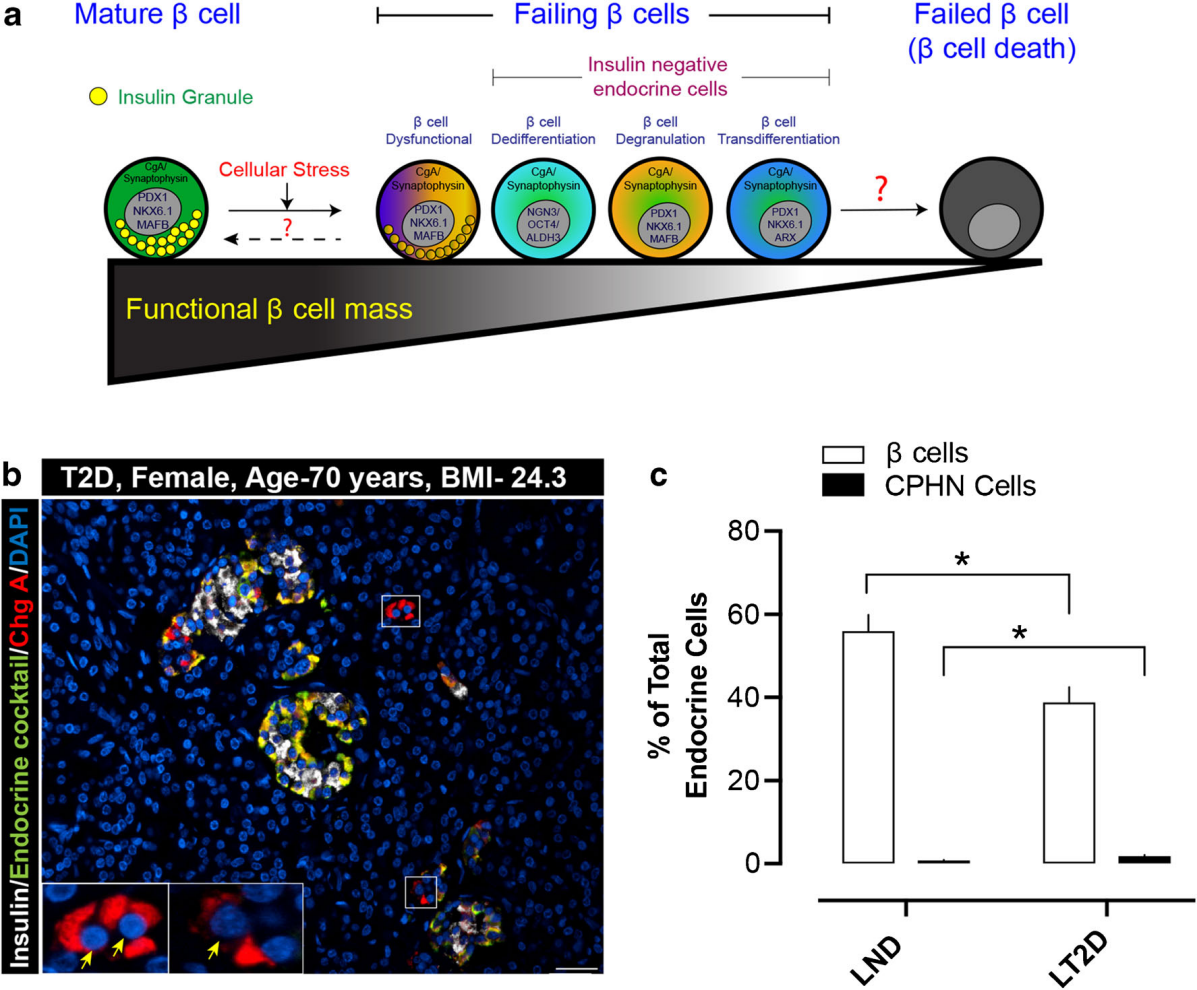
**a** A schematic illustrating the possible “altered differentiation” states of human pancreatic β cells. In response to cellular stresses (glucotoxicity, ER stress, immune attack or viral infection), β cells can undergo functional or morphological changes. These alterations might include insulin-positive dysfunctional β cells or insulin-negative β cells that are dedifferentiated (expressing transcription factors of endocrine progenitor cells), degranulated (“empty” β cells no longer harboring a normal complement of insulin granules), or transdifferentiated (transitioning towards a different endocrine cell subtype). At this stage, the β cells may be recognized as “failing β cells,” as they do not contain any releasable insulin. The unanswered questions are whether human β cells from those transition states can revert back towards the mature, fully functional state, or whether they will eventually fail completely and undergo apoptotic death. **b** Example of chromograninA-positive hormone-negative (CPHN) cells in the pancreas of a lean subject with type 2 diabetes (Age, 70 years, BMI, 24.3). The merged immunofluorescent image is a selected field of 4-μm paraffin section of pancreas stained for insulin (white), endocrine cocktail (glucagon, somatostatin, pancreatic polypeptide and ghrelin) (green), chromograninA (ChgA) (red), and DAPI (blue). The cells staining only for chromograninA and not for any of the known pancreatic hormones (CPHN cells) are therefore stained red (and indicated by yellow arrows). Inset, high-power image of the selected area (marked by white square in the low power image) indicating the CPHN cells. Scale bar, 50 μm. **c** Percent of β cells and CPHN cells (of total endocrine cells) in lean human subjects with no diabetes (LND) and lean human subjects with type 2 diabetes (LT2D). Data are presented as mean ± SEM, *N* = 10 (each group). *, *p* < 0.01

Dedifferentiation of a cell is determined by either a reversal of the differentiation signature back towards a progenitor-like state or loss of terminally differentiated markers and phenotype [65, 66]. β Cell dedifferentiation, however, is a broad and loosely defined term that generally refers to an alteration in β cell phenotype. β Cell dedifferentiation was first reported in rats where prolonged exposure to hyperglycemia correlated with progressive loss of β cell differentiation (as determined by altered expression of several key islet transcription factors and other islet genes important for normal glucose-stimulated insulin secretion [GSIS]) [7]. Further studies in rodents and humans indicated that β cell dedifferentiation manifests as reduced expression of β cell–enriched genes [65, 66]; gradual loss of β cell specific transcription factors including PDX1, MAFA, and FOXO1 [6, 67, 68]; and re-occurance of endocrine progenitor markers (NGN3 and OCT4 [3] or ALDH1 [9], for example). These alterations cause β cells to undergo metabolic and structural reconfiguration, exiting their mature state (“failing β cells”) and ultimately results in defective GSIS (Fig. 1a). What remains undetermined is whether these failing β cells can re-differentiate back into a mature state, remain as dedifferentiated cells with poor ability to secrete insulin in response to glucose, or, alternatively, whether β cell death is inevitable. Therefore, the importance of β cell dedifferentiation stems from the fact that, along with phenotypic cellular changes, there is diminution of the cell’s ability to optimally respond to a glucose challenge with insulin secretion. Dysregulated insulin secretion ensues a well-recognized phenomenon in the pathogenesis of both T1D and T2D, and, together with the established mechanism of β cell apoptosis [2, 69], β cell dedifferentiation has been proposed as a factor in the loss of functional β cells in T1/T2D. [3, 9, 11].

## Transdifferentiation of Pancreatic β Cells—Altered Identity Continued

Transdifferentiation refers to the process, whereby a mature endocrine cell transforms into another cell type without reverting backwards towards a more primitive progenitor-like state. Only a few examples of transdifferentiation of human cells have been reported to date, and those require forced expression of transcription factors or miRNAs for the conversion of human fibroblasts into neurons, hematopoetic progenitors, or brown fat cells [70] or of human liver cells into β cells [71]. Even though mature human β cells are considered as “non-switching” in terms of their hormone production, spontaneous conversion of human β cells into glucagon producing α cells has been reported during islet cell reaggregration in vitro [4]. These results are consistent with other reports where, in both T1D and T2D, β cells transdifferentiated into α cells in humans [10, 72] or into δ cells in rats [73] and mice [13].

Various mouse models of diabetes have also revealed that a percentage of existing β cells can adopt the mature identity of glucagon-producing α cells under hyperglycemic conditions [3, 12]. While it has not been firmly established, it appears that loss of β cell mass/function is the driving force towards this seemingly counterintuitive change. Insulin may be required to maintain β cell identity, a reduction in insulin release in the local islet environment potentially having an impact upon the identity of endocrine cells. Inappropriately increased α cell function and consequent hyperglucagonemia has long been recognized as a contributor to hyperglycemia in diabetic patients, via stimulation of hepatic glucose production [74]; therefore, stringent control over β to α-transdifferentiation (by minimizing β cell plasticity) might be helpful in preventing the progression of diabetes. Transdifferentiation of pancreatic α to β cells has also been reported in a mouse model where overexpression of PAX4 [75] or deletion of *Arx* [76, 77] in α cells resulted in loss of α cells through transdifferentiation to β cells. These data suggest potential new avenues for restoration of β cell mass in diabetes, not only by providing alternative sources of β cells, but also by reducing α cell mass, and thus potentially restoring the insulin-glucagon balance, which is perturbed in diabetes [78, 79]. Apart from α to β transdifferentiation, the regenerative process of endocrine pancreas also includes transdifferentiation of pancreatic ductal cells to endocrine β cells in rodents. By utilizing a genetic lineage-tracing approach (a mouse model used to trace the ductal specific human carbonic anhydrase-II (CA-II)-positive cells in which the CA-II promoter is conjugated with the *Cre-Loxp* system) showed that CA-II-positive cells merged with β cells in the adult pancreas and ligated duct, suggesting a potential transdifferentiation of ductal cells to generate new islets [80]. Moreover, the existence of rat and human pancreatic progenitor cells in the duct and their differentiation potentials has also been reported [81, 82]. The regenerative capacity of endocrine pancreas from ductal sources is also supported by our recent report showing an increased proliferation of the pancreatic duct gland (PDG) compartment in humans with T1D [83•].

## Altered Identity of Pancreatic β Cells in Humans with T1D and T2D—Are β Cells Hidden or Camouflaged?

Circumstantial evidence of an altered β cell phenotype in clinical diabetes was revealed by the observation of co-localization of insulin with glucagon or vimentin (a mesenchymal marker) in a distinct subset of cells in human pancreas sections from subjects with T2D [84, 85]. Likewise, a significant increase in bihormonal insulin^+^/glucagon^+^ and NKX6.1^+^/amyloid^+^/glucagon^+^ cells was found upon analysis of mature β cell markers (e.g., MAFA, FOXO1, NKX6.1) in T2D human and nonhuman primate pancreas [10]; a greater frequency of Nkx6.1^+^ glucagon^+^ insulin^−^ cells was found in islet amyloid-positive regions.

An altered phenotype in islets from subjects with T2D was observed with an ~ 3-fold increase in the number of pancreatic islet cells (insulin^−^/synaptophysin^+^/ALDH1A3^+^ cells) that no longer expressed any of the major pancreatic hormones, yet retained endocrine features, thus implying dedifferentiation of β cells in T2D [9]. Moreover, in T2D, human β cells were found to express gastrin (an embryonic pancreas marker), a phenotype that resolved upon glucose normalization, suggesting reversible β cell reprogramming in T2D [55, 86].

We first reported an increased frequency of endocrine cells that express no known islet hormones but do express the endocrine marker chromograninA (chromogranin A-positive hormone-negative [CPHN cells]) in humans with T1D [87••, 88] and T2D [8••, 89••] (Fig. 1b). CPHN cells occurred within established islets but were most frequently found as scattered cells, either singly or in small clusters in the exocrine pancreas. Despite the lack of expression of any endocrine hormones in CPHN cells in T1D, the presence of β cell–specific transcription factors (for example, NKX6.1 and NKX2.2) found mainly in the scattered or single cells suggested that those cells represent a pool of “hidden β cells” [87••]. While it is potentially plausible that these cells were formerly β cells that have undergone dedifferentiation or transdifferentiation, this is less likely given their distribution and increased frequency as scattered cells in exocrine pancreas in the setting of diabetes; it is therefore possible that they represent partially differentiated, newly formed endocrine cells. In support of this, we further reported abundant scattered CPHN cells in the exocrine compartment of human fetal and neonatal pancreas [8••], the highest frequency being in fetal pancreas compared to neonatal [90], potentially suggesting that the increased frequency of CPHN cells containing the β cell–specific transcription factors NKX6.1 or NKX2.2 in both T1D and T2D may be indicative of attempted β cell regeneration.

This conclusion is consistent with previous findings where pancreatic β cell regeneration in humans with recent-onset T1D was reported [91, 92]. Moreover, persistent residual β cell function in patients with recent-onset or, in some cases, very long-standing T1D has been reported [93–95], with measurable levels of C-peptide detected in serum of donors with long-standing T1D [96•, 97••]. To date, it has not been possible to trace the β cell lineage in those individuals to determine if their residual β cells had (1) dedifferentiated, evaded immune attack, and then redifferentiated again (perhaps once insulin therapy had been initiated) [98]; (2) transdifferentiated from other endocrine cells; or (3) are newly generated β cells in a transition state towards maturity. Whether due to loss of identity or attempted regeneration, the altered endocrine phenotype in T1D/T2D likely reflects an adaptive response (either to camouflage the endocrine cells from potential destruction or to expand/regenerate).

One approach to establish the sequence of events leading to defective β cell function and mass in diabetes is to evaluate the pancreas from prediabetic individuals. However, there was no change in the frequency of CPHN cells in pancreas from nondiabetic autoantibody-positive brain dead organ donors (unpublished data); however, most of these donors had a single autoantibody to a T1D autoantigen, which is associated with low risk of progression to clinical diabetes in living subjects, and they did not have evidence of insulitis or beta cell destruction. Perhaps it would be more instructive to study pancreas from double or triple antibody-positive prediabetic donors to elucidate the pattern of endocrine cell identity alterations in incipient diabetes; these donors are, however, quite rare [99••].

Whilst the evidence reported in the literature from numerous sources supports the concept that dedifferentiation or transdifferentiation of β cells does occur in T1D and T2D, one critical question is to what extent? We found ~ 3% increase in Ins^+^/Glu^+^ bihormonal cells in the pancreas in subjects with T2D with a further increase to about 16% in incretin-treated individuals with T2D [85]. This ~ 3–4% frequency in T2D was later confirmed [10••]. To address the quantitative contribution of altered β cell identity to the β cell deficit, we studied the frequency of polyhormonal cells and determined that changes of β cell identity that have been ascribed to loss of differentiation in T2D could only account for approximately 2% of β cell loss (Fig. 1c) [89••]. In addition, our data revealed an increased frequency of polyhormonal cells (Ins^+^/hormone cocktail^+^ cells) in lean subjects with T2D [89••]. Given that human pancreas, obtained at the time of surgery or at post-mortem, is by definition a single timepoint for any given individual, it is not possible to definitively determine the evolution and fate of those “camouflaged” Ins^+^ β cells in humans with T2D.

## Mechanisms Involved in β Cell Dedifferentiation in T1D and T2D

The exact mechanisms of pancreatic β cell dedifferentiation in humans with T1D or T2D is yet to be elucidated (potential drivers of the β cell dedifferentiation in T1D and T2D are listed in Table 1). Our cross-sectional study in pancreas of humans with T1D demonstrated no T cell infiltration (as adjudged by staining for the presence of CD45) in or around hormone-negative endocrine cells, suggesting that direct interaction of autoimmune T cells with β cells might not be responsible for alterations in β cell identity in humans with T1D; however, it is also possible that CPHN cells are not yet expressing surface antigens that attract autoimmune T cells [87••]. In a different setting of pancreatic pathogenesis, where inflammation plays a central role, we reported increased co-expression of islet-derived chemokine (CXCL10) and chemokine receptor (CXCR3) in Ins^+^/Glu^+^ double-positive cells in humans with chronic pancreatitis [111].

**Table 1:** Drivers of altered identity of β cells in T1D and T2D

Cellular and physiological modulators	Altered identity of β cells in		Most relevant references
	T1D	T2D	
Hyperglycemia	Documented	Documented	[3••, 7, 9••, 10••, 11••, 87]
Inflammation	Possibly linked	Documented	[100–102]
Obesity	Possibly linked,(no direct evidence)	Documented	[8••]
miRNA	Possibly linked (no direct evidence)	Possibly linked	[103]
Epigenetics	Possibly linked	Possibly linked	[104••, 105•, 106•]
Beta cell heterogenity	Documented	Documented	[57, 58]
Viral infection	Possibly linked	Possibly linked (no direct evidence)	[107]
Protein misfolding/ER stress	Possibly linked	Possibly linked	[108–110]

Islet-derived proinflammatory mediators might induce β cell dedifferentiation in human T1D, as the islet micoenvironment has also been reported to be the generator of proinsulin targeting CD4 T cells in humans with T1D [100]. Such impaired “self-maintenance” of pancreatic β cells has also been observed in T2D, where cytokines and chemokines (secreted by β cells) recruit macrophages by inducing stress signaling in islets [101], and this may ultimately play a role in “inflammation-induced dedifferentiation” in T2D [102••]. The molecular mechanism of such β cell decompensation is not known, however though, factors such as microRNAs [103] or virus-like infection (enteroviral infection) [107] might be key regulators of this process.

Extensive animal and in vitro studies have implicated glucotoxicity and dysregulation of downstream pathways as being involved in β cell dedifferentiation. Cellular events, like hyperglycemia-induced alterations of β cell–specific gene expression (for example, downregulation of β cell–enriched genes and upregulation of β cell “forbidden” genes) (extensively reviewed in [112••]), might lead to the altered phenotypic and functional changes that are the hallmarks of a dedifferentiated β cell. Despite the fact that high glucose levels, as well as the duration of hyperglycemia, are two major upstream regulators of β cell dedifferentiation, our experimental data has demonstrated that the increase in both CPHN cells and polyhormonal endocrine cells in the human IAPP-transgenic (HIP) rat (a model of T2D) is already present by 2–3 months of age, thereby preceding diabetes onset, elevations in glucose, or any measurable loss of β cell mass [89]. This implies that β cell stressors, in the HIP rat consequent upon IAPP mis-folding and cell dysfunction induced by IAPP-derived toxic oligomers, provoke the alteration in β cell identity and the increase in CPHN cells, rather than the hyperglycemia that develops only from the age of 5 months in this model [113]. Peptide-based analyses of epitope targeting by either CD4 or CD8 T cells revealed a plausible role of IAPP as an autoantigen in the pathophysiology of T1D both in humans and in a non-obese diabetic (NOD) mouse model of spontaneous autoimmune diabetes [114–117]. Recently, histological studies on pancreas from subjects with recent onset T1D have revealed that amyloidosis is also present in T1D [118, 119•].

Endoplasmic reticulum (ER) stress with inadequate induction of an adaptive unfolded protein response (UPR) might be a key driver of β cell dedifferentiation. β Cells are extremely dependent on their ER to cope with the oscillatory requirement of secreted insulin to maintain normoglycemia. Altered metabolic states, for example in obesity, result in decreased insulin sensitivity in the skeletal muscle, liver, and adipose tissues that is counteracted by a compensatory increase in insulin secretion by β cells through an increase in both β cell function and mass [120]. For insulin translation and folding, β cells rely greatly on the unfolded protein response (UPR), an array of three main signaling pathways designed to maintain ER homeostasis and limit ER stress [121]. A recent study demonstrated that targeted deletion of ATF4 (the main transcriptional regulator of UPR) in β cells in Akita mice (*Akita/βATF4KO*) leads to the increase in number of glucagon, somatostatin, or pancreatic polypeptide-positive cells in islets, suggesting increased β cell dedifferentiation [108]. Therefore, the fine-tuning of this adaptive UPR is vital for the preservation of the β cell differentiated phenotype and failure of this process is associated with the progression to diabetes and altered β cell differentiation [109, 110].

Epigenetic regulation of pancreatic β cell identity could also play a crucial role in maintaining β cell plasticity. For example, the α cell–specific DNA-binding protein *Arx* must be repressed to prevent β- to α-transdifferentiation in a mouse model [122]. *Arx* silencing in β cells is accomplished, at least in part, by binding of the transcription factor Nkx2.2 to the *Arx* promoter, followed by recruitment of DNA methyltransferase Dnmt3, increased DNA (CpG) methylation, and finally binding of MeCP2, a DNA-binding protein, to the promoter region of *Arx* in βcells to establish and maintain a fully repressed state [104••, 123]. Recently, unbiased epigenome mapping and single-cell RNA sequencing (ScRNA-Seq) have revealed that the β cell–specific chromatin regulatory system, Polycomb (Eed/PRC2), is necessary for maintenance of global silencing and terminal differentiation of β cells in the mouse [105•]. Therefore, as observed in rodent models of diabetes [55], it is likely that epigenetic dysregulation similarly contributes to β cell dedifferentiation in humans with T1D or T2D [106•].

## Concluding Remarks and Future Perspectives

Pancreatic β cell dedifferentiation encompasses both a loss of one or more elements that are present in a mature functional β cell (this occurring for a multitude of reasons, including cellular stress or senescence) and a regression back towards a more primitive state, such as the pluripotent progenitor cell from whence it came.

Emerging evidence suggests that altered β cell identity in humans with T1D and T2D contributes to impaired β cell function in diabetes; however, the exact mechanisms are not known. Such cellular adoption of a resting or dedifferentiated state for a subset of pancreatic β cells might be a natural phenomenon that temporarily allows interruption of regular cellular function, serving as a protective mechanism to circumvent damage or death.

In order to derive benefits from the inherent plasticity of pancreatic β cells in humans with T1D or T2D, future research should aim to enhance our understanding of some key questions. Firstly, the extent of altered β cell identity in humans with prediabetes is yet to be determined, and this knowledge would be instructive in characterizing β cell dedifferentiation in humans. Secondly, it is imperative to identify key regulatory molecules and molecular markers of β cell dedifferentiation in humans (possibly by using laser capture microdissection of the altered β cell followed by single-cell RNA-seq). Thirdly, it is crucial to understand how the expression of chromograninA remains unaltered in the dedifferentiated β cell, given that insulin and chromograninA reside in the same dense-core granules of the β cell. Finally, effective re-differentiation mechanisms directed towards “altered state” pancreatic β cells should be explored, as identifying ways to inhibit or reverse these stages could substantially enhance the scope for developing novel therapies for restoration of β cell function in diabetes.
